# Supplementary material for: Identification of photoperiod-induced specific miRNAs in the adrenal glands of Sunite sheep (Ovis aries)
Source: Front Vet Sci. 2022 Jul 22;9:888207. doi: 10.3389/fvets.2022.888207 (PMC9354845; doi:10.3389/fvets.2022.888207)
Supplement: Supplementary file 1 [file Data_Sheet_1.ZIP › Supplementary file/miRDeep2 file.zip!/miRDeep2 file/Know_Structure/LP42A1/oar-mir-133.pdf]

miRBase precursor : oar-mir-133
Total read count : 571
oar-miR-133 read count : 553
remaining reads : 18

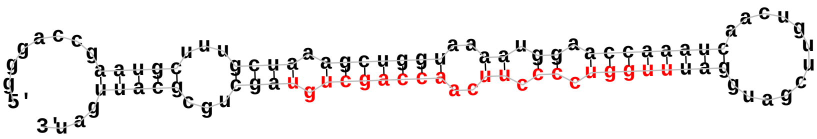

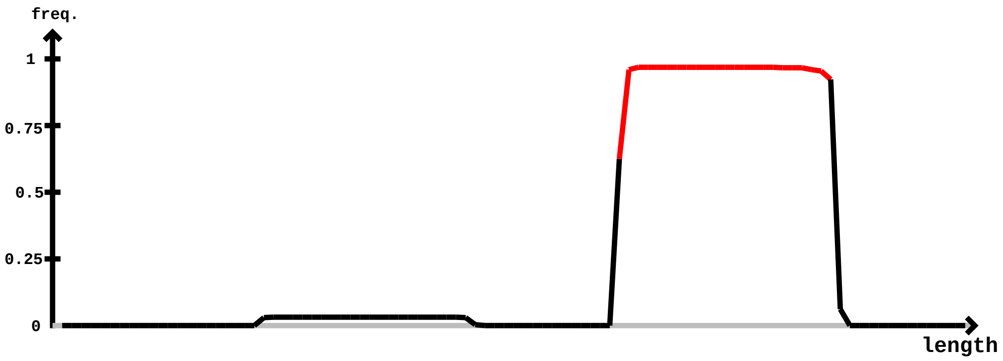


```
                                                         oar-miR-133
5'- gggaccgaaugcuuugcuaaagcugguaaaauggaaccaaaucaacuguucgauggauuugguccccuucaaccagcuguagcugcgcauugau  -3'    exp
    .......(((((...((((.(((((((..((.((.((((((((............)))))))).)).))..))))))).))))...)))))...   reads   mm   sample
    ....................agcugguaaaauggaaccaaa.....................................................   1       0    seq
    ....................agcugguaaaauggaaccaaau....................................................   15      0    seq
    ....................agcugguaaaauggaaccaaauc...................................................   1       0    seq
    .....................gcugguaaaauggaaccaaauc...................................................   1       0    seq
    .........................................................uuugguccccuucaacU....................   1       1    seq
    .........................................................uuugguccccuucaaccagc.................   4       0    seq
    .........................................................uuugguccccuucaaccagcu................   3       0    seq
    .........................................................uuugguccccuucaaccagcug...............   15      0    seq
    .........................................................uuugguccccuucaaccagcuA...............   2       1    seq
    .........................................................uuugguccccuucaaccagcugu..............   321     0    seq
    .........................................................uuugguccccuucaaccagcugua.............   11      0    seq
    ..........................................................uugguccccuucaaccagcug...............   1       0    seq
    ..........................................................uugguccccuucaaccagcugu..............   167     0    seq
    ..........................................................uugguccccuucaaccagcugua.............   23      0    seq
    ...........................................................ugguccccuucaaccagcugu..............   4       0    seq
    ...........................................................ugguccccuucaaccagcugua.............   1       0    seq
```
